# Simultaneous Bilateral Total Hip Arthroplasty with Straight-Stems and Short-Stems: Does the Short One Do a Better Job?

**DOI:** 10.3390/jcm12031028

**Published:** 2023-01-29

**Authors:** Patrick Reinbacher, Andrzej Hecker, Joerg Friesenbichler, Maria Smolle, Lukas Leitner, Sebastian Klim, Alexander Draschl, Danijel Colovic, Kevin Brunnader, Andreas Leithner, Werner Maurer-Ertl

**Affiliations:** 1 Department of Orthopaedics & Traumatology, Medical University of Graz, Auenbruggerplatz 5, 8036 Graz, Austria; 2 Division of Plastic, Aesthetic and Reconstructive Surgery, Department of Surgery, Medical University of Graz, Auenbruggerplatz 5, 8036 Graz, Austria

**Keywords:** total hip arthroplasty, short-stem, straight-stem, one-stage, bilateral

## Abstract

Background: Total hip arthroplasty (THA) is known to be the most successful orthopaedic surgery of the last century, but it is still struggling with controversies concerning one-stage bilateral THA. The current study aimed to compare the clinical outcome of patients with unilateral or simultaneous bilateral THA by using short-stem and straight-stem designs and focusing on operation time, blood loss, and length of hospital stay (LOS). Material and Methods: Between 2006 and 2018, 92 patients were enrolled in this study. Forty-six patients underwent a bilateral THA in one session, and forty-six matched patients underwent a unilateral THA. In each of the two groups (unilateral vs. bilateral), 23 patients received either a straight (unilateral: 10 females, 13 males, mean age 63; bilateral: 12 females, 11 males, mean age 53 years) or short stem (unilateral: 11 females, 12 males, mean age 60 years; bilateral: 12 females, 11 males, 53 mean age 62 years). The blood count was checked preoperatively as well as one and three days after surgery. Furthermore, the operation time and LOS were investigated. Results: Compared to THA with straight-stems, short-stem THA showed significantly less blood loss; there was no difference in the LOS of both groups. A significantly shorter operative time was only observed in the bilateral THA. Conclusion: The current study showed that simultaneous bilateral THA appears to be safe and reliable in patients without multiple comorbidities. In addition, short-stem THA appears to be beneficial in terms of clinical performance and outcome, and it appears to be superior to straight-stem THA, regardless of whether the patient underwent unilateral or simultaneous bilateral THA.

## 1. Introduction

Total hip arthroplasty (THA) is widely known as “the orthopaedic surgery of the last century”. Furthermore, it is one of the safest and most cost-effective orthopaedic surgical procedures. Since the first hip replacements in the early 1960s, surgical techniques and implants have significantly improved. Furthermore, THA has developed from a geriatric surgery to a lifestyle surgery, with increasing numbers of operations being performed on younger and physically more active patients [1,2,3,4]. Therefore, the requirements for implant survival, durability, and functional outcomes in terms of daily activities and sports continue to increase. Primary unilateral THA is a very successful operation that improves patient quality of life and is associated with low complication rates. However, some previous studies have shown that patients undergoing unilateral THA may require contralateral surgery within 1–10 years [5,6,7,8,9]. In addition, if both hips are affected simultaneously, bilateral THA may be necessary and might also be of interest to younger patients with concerns about loss of work. The advantages of one-stage bilateral THA include that patients only need to undergo anaesthesia once and only have one hospital stay, resulting in a shorter length of hospital stay (LOS) as well as a reduction in cost compared with two-staged bilateral procedures [1,3,6,7,10,11,12,13].

On the other hand, several opposing studies have shown that one-stage bilateral THA poses greater risks to patients, such as higher transfusion rates due to increased blood loss, more adverse events, and suboptimal functional outcomes [6,9,10,12,14,15,16]. However, Donner et al. [5] have reported that patients with one-stage bilateral short-stem THA were highly satisfied with their sports and recreational activity at mid-term follow-up. Bilateral one-stage THA offers the advantage of the patient being prosthetically treated with respect to the replacement of both hip joints after one session if their physical health allows for it [17,18,19,20]. Furthermore, by having a shorter LOS, bilateral one-stage THA is beneficial in reducing overall hospital costs compared with two-stage bilateral THA [17,18,19,20,21]. Taking into account the advantages of one-stage bilateral THA as mentioned above, according to Micicoi et al., bilateral THA in one operative session is recommended for ASA 1 and 2 patients, aged under 80 years with disabling bilateral osteoarthritis [20].

Short-stem THA was introduced in the 1990s to preserve proximal femoral bone stock, prevent proximal stress shielding, guarantee better functional outcomes due to more physiologic biomechanical properties, and increase the survival rates of the implants [1,5,22]. Another advantage of short-stem THA is the more accessible application of minimally invasive approaches [3]. However, there is a large variety of different short-stem designs available on the market, and owing to their design, they are reduced in length and diaphyseal fixation compared with straight-stems, which has raised concerns regarding higher rates of aseptic loosening and, consequently, revision rates [23,24,25,26]. Moreover, short-stems have shown a tendency towards early distal migration, which might occur due to their mainly metaphyseal anchorage and smaller bone–implant interface [2,24,27,28]. Hauer et al. [2] reported low revision rates with satisfying results when comparing different types of short-stem devices in a comparative analysis of 52 studies. Schnurr et al. [29] published the revision rates of short-stem designs that were comparable to traditional cementless straight-stem designs. Therefore, with short-stems showing excellent short- to mid-term results, traceable early subsidence may be interpreted differently from straight-stems [28].

The current study aimed to report our clinical experience with simultaneous bilateral THA with two different stem designs by comparing a short-stem with a straight-stem. We focused on the transfusion rates, complications, LOS, and early readmission rates. Additionally, we compared the clinical findings of patients having simultaneous bilateral THA procedures with matched patients having unilateral THA procedures.

## 2. Materials and Methods

In this study, a retrospective comparative analysis was performed on 92 prospectively included patients. For this purpose, the institutional database was reviewed for patients who had received unilateral or simultaneous bilateral THA at the Medical University of Graz, Austria (Department of Orthopaedics & Traumatology) between 2006 and 2018. The present study was approved by the Ethics Committee of the Medical University of Graz, Austria (Ethical Committee No. 28-152 ex 15/16).

As per standard procedure, an anterolateral approach to the hip was performed by two experienced orthopaedic surgeons at a single university hospital. All patients received uncemented components. The cementless straight-stem (Corail^®^ Hip System, DePuy International Ltd., Leeds, England, UK) was regularly used for more than 10 years at our department before the short-stem with mainly metaphyseal fixation (ANA.NOVA^®^ Alpha Schaft^®^ Proxy, ImplanTec GmbH, Moedling, Austria) was introduced in 2016. The characteristics of the two stems have been previously illustrated by our research group [30]. In general, the more symptomatic hip joint was operated on first in the case of bilateral THA, and no operations were aborted because of intraoperative complications. After completing the first hip, a sterile dressing was applied, and the second hip joint was prepared with the same instruments. An intraoperative cell saver was not used routinely, and no drains were used postoperatively. No tranexamic acid was used perioperatively. Full weight bearing using two crutches was allowed in all cases postoperatively, prophylaxis against deep vein thrombosis was administered for 6 weeks, and patients had to wear stockings against deep vein thrombosis (DVT). Usually, the patients were discharged home, and older patients or patients who could not go home were transferred to geriatric mobilisation units.

The patients’ hospital records, rehabilitation discharge summaries, and follow-up office notes were used for the analysis. The data on blood loss were collected from the available hospital records, and the blood count was checked preoperatively as well as one day and three days post-operation. Furthermore, the clinical documents were reviewed to administer allogenic blood products. The operation time was documented and collected from the surgical reports. The length of stay (LOS) was also gathered from the clinical reports and were calculated from the day before the operation until the day of discharge. The major complications were death, pulmonary embolism (PE), DVT, cardiovascular and/or pulmonary complications, neurological complications, and complications associated with the implant needing revision surgery.

SPSS Statistics program 25 (SPSS Inc., Chicago, IL, USA) was used for the statistical analysis. The Shapiro–Wilk test was used to test the normality of the data. For the demographic data, a Student’s *t*-test for independent samples was chosen to determine the statistical differences in the parametric data; in the non-parametric data, the Mann–Whitney U-test was used. A one-way ANOVA test was used to compare continuous datasets, and the Bonferroni post hoc test was subsequently used when the significant main effects were present. All of the statistical tests were two-tailed, and the differences were considered to be statistically significant when *p*  <  0.05. A post hoc power analysis was calculated according to Hoenig and Heisey for the magnitude of differences in all compared parameters [31]. The selected sample size per group was sufficient for a statistical analysis.

## 3. Results

All demographic data between patient groups were quite similar; only the patients in the bilateral short-stem group were significantly older than the straight-stem group (Table 1). Furthermore, significant differences were observed for the follow-up due to the fact that the short-stem was introduced in 2016.

### 3.1. Straight-Stem Groups

Forty-six patients underwent THA between 2006 and 2017 and received a straight-stem design (Corail^®^ Hip System, DePuy International Ltd., Leeds, England, UK). Meanwhile, 23 patients had a unilateral procedure, and 23 patients had a one-stage simultaneous bilateral THA. There were 11 male and 12 female patients with a mean age of 53 years at the time of operation (range, 25–88 years) in the simultaneous bilateral THA group. In the unilateral THA group, there were 12 male and 11 female patients, with a mean age of 63 years (range, 38–85 years).

### 3.2. Short-Stem Groups

Forty-six patients underwent THA between 2016 and 2018, receiving a short-stem THA (ANA.NOVA^®^ Alpha Schaft^®^ Proxy, ImplanTec GmbH, Moedling, Austria) with a novel implant design. These patients were also enclosed in an ongoing prospective clinical surveillance study. In the bilateral group, there were 11 male and 12 female patients, with an average age of 62 years (range, 38–78 years). In the unilateral short-stem group, there were 13 male and 10 female patients, with a mean age of 60 years (range, 46–72 years).

The bilateral short-stem group showed a significantly shorter operation time and less blood loss. The unilateral short-stem group showed no significantly shorter operation time but significantly less blood loss. However, no difference was found in the LOS when compared with straight-stem THA patients (Table 1).

Within the first three days after surgery, five patients in the bilateral and three patients in the unilateral straight-stem group received allogenic blood products (two units per patient) due to a lowered red blood count and suffered from clinical symptoms such as low blood pressure, dizziness, or reduced general condition. In the same time period, another three patients in the bilateral short-stem group also received blood products (two units per patient) for the same indications.

Overall, there was no history of myocardial infarction, deep vein thrombosis, pulmonary embolism, or death associated with the surgical procedure during follow-up. Furthermore, there was no postoperative readmission to the hospital in the short-term (30 days) for any medical or surgical reason for neither the straight-stem nor short-stem groups.

Two revisions had to be performed in the bilateral straight-stem group (overall complication rate: 2.1%), the first one being due to increased serum metal ions concentrations after the usage of a metal-on-metal bearing 57 months after implantation; the second one was due to a low-grade periprosthetic joint infection (PJI) with *Staphylococcus epidermidis* 32 months after the index procedure. The PJI was treated with a one-stage procedure and antibiotics; the patient is doing well 16 months after revision surgery. One patient from the straight-stem group died 32 months after index surgery due to chronic renal failure with dialysis.

## 4. Discussion

The current study revealed satisfying results with low complication rates in all implant groups and in both the bilateral and unilateral groups. Overall, patients with short-stem THA showed better clinical data with respect to operation time, blood loss, and LOS, although these differences were not statistically significant.

In the literature, there are still controversies regarding the advantages and disadvantages of one-stage or two-stage bilateral THA, and there are no actual recommendations from orthopaedic societies [1,4,6,8,9,10,11,12,14,29,32,33]. The decision and indication for the single-stage bilateral procedure and the selection of the appropriate patient remain the responsibility of the orthopaedic surgeon. Most studies reporting one-stage bilateral THA were performed in dedicated centres with similar effectiveness and morbidity for unilateral or two-stage bilateral THAs. Many previous studies have reported outcome and complication rates comparing one-stage and two-stage THA procedures; in a smaller number of studies, the surgical approach was also analysed as an influencing factor [3,12]. The current study’s aim was to compare one-stage and two-stage THA procedures using different stem types regarding the outcome and complication rates.

The meta-analysis of Shao et al. [8] showed that one-stage bilateral THA had a lower risk of major systemic complications, less deep venous thrombosis, and shorter operative time compared with two-stage bilateral THA; Guo et al. [6] and Charity et al. [1] recently confirmed these observations as well. There were no major complications in the current series, which might be explained by the small number of patients enrolled. Furthermore, in most studies, patients receiving simultaneous bilateral THA are more so selected with respect to demographics and comorbidities. In all implant groups in the current series, the distribution of comorbidities was equal with respect to the ASA score (Table 1). Additionally, improvements in anticoagulation therapy have significantly reduced the rate of deep venous thrombosis and pulmonary embolism.

Aghayev et al. [32], Parvizi et al. [15], and other studies have related low complication and morbidity rates in one-stage bilateral THA with the higher numbers of patients included [1,6,12]. Stavrakis et al. [9] have reported that the overall risk for complications following bilateral THA was similar to that observed after unilateral procedures; on the other hand, higher rates of septic complications were reported in the bilateral group. As short stems offer the possibility of revision surgery with conventional straight stems, using short stems in bilateral THA could be clinically beneficial in the long term for surgical management, especially for patients under the age of 65 years, who are known to be at higher risk for surgical revision in the future [23,34,35,36,37]. However, there is a lack of long-term results regarding the effect of short-stems’ observed early subsidence on implant survival [28]. Therefore, long-term studies are needed to assess the axial migration’s impact on implant survival, as short stems seem to be a promising alternative to straight stems in simultaneous bilateral THA.

Previous studies have described the age at the time of surgery as an important factor influencing the outcome of one-stage bilateral procedures. However, in the current series, the mean age of patients receiving simultaneous bilateral THA using a short-stem device was significantly higher than the straight-stem group. Still, no higher complication rates were observed, which is novel when compared with the literature.

Short-stem THA procedures showed significantly shorter operation times in the bilateral group as well as the unilateral group. Several other studies comparing the operative time between one- and two-stage bilateral THA procedures have demonstrated that the one-stage bilateral procedure takes less surgical time than the two-stage procedure [1,3,6,8,12,15]. Moreover, it has been reported that using a conventional rather than a short stem is a significantly influential factor regarding longer operative time [38]. Furthermore, Surace et al. [39] have suggested a strong correlation between increased operative time and perioperative complications in primary short-stem THA. Additional factors such as the surgeon’s experience and routine will also positively influence the theatre time and influence the use of gentler and bone-sparing operation techniques in short-stem designs. A shorter operation time might result in reduced blood loss, lower transfusion rates, and earlier postoperative mobilisation due to missing fatigue complications.

Parvizi et al. [4] found no statistically significant difference in the 90-day mortality between unilateral and bilateral THA in a prospective matched study, although the simultaneous bilateral THA group required more blood transfusions and showed lower haemoglobin concentrations upon discharge than the unilateral group. The current series also showed a low transfusion rate of 12% (11 out of 92 patients); eight transfusions had to be performed in the simultaneous bilateral groups, and three had to be performed in the unilateral straight-stem group. Nevertheless, no major cardiovascular complications or increased infection rates were observed in patients receiving allogenic blood products.

Besides functional outcome and complication rates following THA surgery, the postoperative hospital costs and LOS are important factors. Several studies have indicated shorter stays and lower costs in one-stage bilateral procedures than in the two-stage bilateral THA [3,6,8,10,13,15]. In the current series, the LOS was shorter in the bilateral short-stem group compared with the straight-stem group, although the difference was statistically insignificant. In the unilateral group, the LOS was equal, which contrasts the findings of Hauer et al., who have reported a significantly shorter LOS for the unilateral short-stem THA compared with the unilateral straight-stem THA—although the study was conducted in a younger study population—but without a significant difference in the return to work time, indicating that the stem design does not influence the recovery time [40]. Lorenze et al. [11] and Reuben et al. [13] reported a 25% cost savings in the simultaneous bilateral THA group because the majority of cost reduction can be attributed to the decreased overall LOS. Taking this into account, with a lower LOS for short-stem THA [40,41], total hospital costs can be even more reduced. Recently, Villa et al. [16] reported longer LOS in patients with simultaneous bilateral THA through a direct anterior approach (DAA), which was not proven by Kamath et al. [42] or Parcells et al. [43].

## 5. Limitations

One limitation of the current study is the small number of patients enrolled; on the other hand, we provided matched control groups for both implant types. Another limitation might be the different follow-ups concerning the determination of implant survival. Still, the shorter follow-up of the short-stem groups did not influence the outcome that evaluated the short-term readmission rate. Additionally, the surgical techniques and skills of the operating surgeons increased over time, which might be beneficial for operation time, re-transfusion rates, complications, LOS, and early readmission rates, which makes it more challenging to assess and compare the outcomes. Furthermore, no clinical scoring systems were available to quantify and report the functional results. Given the limitations mentioned above, our results should be mainly interpreted from a health economics point of view.

## 6. Conclusions

The current series showed that simultaneous bilateral THA operation appears safe and reliable in selected patients without multiple comorbidities. In addition, short-stem THA appears to be beneficial in terms of clinical performance and outcome over straight-stem THA, whether unilateral or simultaneous bilateral implantation. Nevertheless, further studies are necessary to confirm these observations.

## Figures and Tables

**Table 1:** Demographic and clinical data.

	Group I (Straight-Stem Bilateral)	Group III (Short-Stem Bilateral)	*p*-Value	Group II (Straight-Stem Unilateral)	Group IV (Short-Stem Unilateral)	*p*-Value
Age at OP, mean (range)	53 (25–88)	62 (38–78)	0.023 ^a^	63 (38–85)	60 (46–72)	0.339 ^a^
ASA score, mean (range)	2 (1–4)	2 (1–3)	0.750 ^b^	3 (1–4)	2 (1–3)	0.152 ^b^
BMI mean (range)	25 (17–39)	26 (18–36)	0.634 ^a^	28 (24–49)	29 (17–36)	0.505 ^a^
Operation time (min), mean (range)	115 (48–254)	68 (51–103)	<0.001 ^c^	55 (38–82)	41 (27–56)	0.303 ^c^
Hb (g/dL) preop., mean (range)	13.2 (9.5–15.6)	14.4 (11.1–16.6)	0.015 ^c^	14.1 (12.0–16.5)	14.4 (12.3–16.3)	1.000 ^c^
Hb (g/dL) day 1 postop., mean (range)	8.8 (5.5–11.1)	10 (7.4–13.1)	0.060 ^c^	10.8 (7.9–14.7)	11.3 (9.4–13.7)	1.000 ^c^
Hb (g/dL) day 3 postop., mean (range)	9.0 (7.3–11.5)	9.5 (7.4–11.4)	1.000 ^c^	10.3 (5.7–13.5)	10.8 (8.1–12.9)	0.475 ^c^
LOS (days), mean (range)	9 (5–17)	8 (4–11)	0.653 ^c^	7 (4–13)	7 (4–10)	1.000 ^c^
Follow-up (months), mean (range)	87 (29–164)	31 (24–49)	<0.001 ^c^	103 (62–137)	49 (47–51)	<0.001 ^c^

^a^ Unpaired *t*-test, ^b^ Mann–Whitney U test, ^c^ one-way ANOVA test. OP: operation; ASA: American Society of Anesthesiologists; BMI: body mass index; Hb: hemaglobin; preop: preoperative; postop: postoperative; LOS: length of stay.

## Data Availability

Detailed data supporting the results are available from the authors.
